# An Illumina metabarcoding pipeline for fungi

**DOI:** 10.1002/ece3.1107

**Published:** 2014-06-02

**Authors:** Miklós Bálint, Philipp-André Schmidt, Rahul Sharma, Marco Thines, Imke Schmitt

**Affiliations:** 1 Biodiversity and Climate Research Centre, Senckenberg Gesellschaft für NaturforschungSenckenberganlage 25, 60325, Frankfurt/Main, Germany; 2 Institut für Ökologie, Evolution und Diversität, Goethe Universität FrankfurtMax-von-Laue-Str. 13, 60438, Frankfurt/Main, Germany

**Keywords:** community ecology, data pruning, high throughput, internal transcribed spacer rDNA, next-generation sequencing

## Abstract

High-throughput metabarcoding studies on fungi and other eukaryotic microorganisms are rapidly becoming more frequent and more complex, requiring researchers to handle ever increasing amounts of raw sequence data. Here, we provide a flexible pipeline for pruning and analyzing fungal barcode (ITS rDNA) data generated as paired-end reads on Illumina MiSeq sequencers. The pipeline presented includes specific steps fine-tuned for ITS, that are mostly missing from pipelines developed for prokaryotes. It (1) employs state of the art programs and follows best practices in fungal high-throughput metabarcoding; (2) consists of modules and scripts easily modifiable by the user to ensure maximum flexibility with regard to specific needs of a project or future methodological developments; and (3) is straightforward to use, also in classroom settings. We provide detailed descriptions and revision techniques for each step, thus giving the user maximum control over data treatment and avoiding a black-box approach. Employing this pipeline will improve and speed up the tedious and error-prone process of cleaning fungal Illumina metabarcoding data.

## Introduction

Metabarcoding rapidly gains importance in ecology, especially in the ecology of microorganisms that are often identifiable only by molecular tools. High-throughput metabarcoding provides unprecedented insights into the composition of these cryptic communities (Bik et al. 2012). The opportunities provided by next-generation sequencing were rapidly embraced in prokaryotic and fungal ecology. Prokaryotes in general are more intensely studied than fungi, but arguably fungi play major roles as symbionts, pathogens, or decomposers in natural and managed ecosystems. For example, fungi play a more dominant role in forest litter cellulose decomposition than bacteria (Stursová et al. 2012). Beyond playing a key role in leaf litter decomposition, fungi may have a knock-on effect on other microbes and subsequent carbon cycling in freshwaters (Frossard et al. 2012). Along with prokaryotes, living fungi are discovered in the most hostile environments, for example, in millions of years old deep-sea sediments (Orsi et al. 2013). Fungi are also highly diverse, with over 1.5 million estimated species worldwide, most of which are currently not described (Hawksworth 2001). It is expected that the field of fungal ecology will strongly benefit from future advances in molecular methods, such as the high-throughput sequencing technologies, which are fundamental for investigating fungal communities.

Data processing currently is a bottleneck in metabarcoding projects. The number of reads per study has been continuously increasing since the introduction of next-generation sequencing (NGS) methods, and it is expected to rise as sequencing technologies advance. Data processing must consider the peculiarities of the taxonomic marker, the sequencing instrument and chemistry, as well as the experimental needs, such as the requirements for sample multiplexing. Well-established pipelines are available to process metabarcoding data (RDP – Cole et al. 2009; MOTHUR – Schloss et al. 2009; QIIME – Caporaso et al. 2010; PANGEA – Giongo et al. 2010; WATERS – Hartman et al. 2010; CANGS – Pandey et al. 2010). However, these tools were mostly developed with the demands of prokaryotic metabarcoding in mind, and it is not straightforward to use them for the specific requirements of fungal metabarcoding, although limited accommodations for fungi already exist (e.g., QIIME). This is partly due to the peculiarities of the designated fungal barcode (Nilsson et al. 2010; Schoch et al. 2012): the internal transcribed spacer (ITS) contains hypervariable and highly conserved regions and is phylogenetically noninformative in distantly related taxa. During data analysis, it is recommended to separate variable ITS regions from the surrounding conserved regions, as conserved regions may distort BLAST assignments (Nilsson et al. 2010). There are pipelines that are specifically suitable for fungal ITS metabarcoding, for example CLOTU (Kumar et al. 2011), SCATA (http://scata.mykopat.slu.se/), PLUTOF (Abarenkov et al. 2010b). However, all of these were developed for 454-sequenced amplicons, and not for other platforms. Further, these pipelines are provided as web services, limiting the users' possibilities to modify them according to specific experimental needs. Pipelines benefit from being rapidly adjustable to keep track with fast developments in data handling techniques (e.g., designation of operational taxonomic units, OTUs – Edgar 2013). Adapting the published complex and multifunctional pipelines for fungal metabarcoding to unconventional sequencing platforms requires not only a deep understanding of their functioning but also substantial programming skills. Data pruning approaches that are not tailored to the users' barcoding marker, sequencing instrument and experimental approach can supply only a suboptimal raw sequence cleanup.

Most metabarcoding studies on fungi have employed 454 pyrosequencing to date. However, recent developments suggest that alternative high-throughput platforms might be more suitable for answering questions in fungal community ecology. This is mainly due to the high read numbers that allow thorough replication (Schmidt et al. 2013). With 454, it is problematic to analyze complex biological samples at sufficient sequencing depth, and at the same time ensure massive sample replication required in microbial ecology (Prosser 2010). In our opinion, the following criteria should be considered when selecting a sequencing platform: (1) resulting reads need to be long enough to contain sufficient variation; (2) both sequence ends should be labeled with the same label, and both of these labels need to be sequenced to avoid tag switching (Carlsen et al. 2012; Lindahl et al. 2013); and (3) the sequencer should allow extensive sample replication, so many samples can be multiplexed in the same run. Furthermore, read numbers should be sufficiently high in each sample, and distributed relatively evenly among samples. We found that the Illumina MiSeq platform satisfies these needs at a low cost, and it is thus a viable alternative to 454. The IonTorrent platforms may also be suitable candidates for replacing the 454 in the future, but Brown et al. 2013 report high error rates in the primer region of the reads, where the multiplexing nucleotide labels are located. Illumina technology does not yet allow for obtaining the entire ITS region (ITS1, 5.8S, and ITS2) with overlapping paired-end reads. Currently, the longest reads are 2 × 300 bp. This allows for the simultaneous sequencing of ITS2 and ITS1 of most species, but without the possibility to overlap these reads in the 5.8S region.

After generating one of the first fungal metabarcoding data sets on an Illumina MiSeq (Schmidt et al. 2013; similar studies Bokulich et al. 2013; McGuire et al. 2013), we found that existing pipelines were not readily usable with our data. It takes a long time to establish a new pipeline from scratch for a new sequencing platform; it is not trivial to determine the optimal number and order of steps; it is important to understand each step/program and evaluate their potential shortcomings, as all scripts and programs might contain programming errors. Finally, the methods applied in pruning metabarcoding data develop rapidly, and there new techniques need to be incorporated into the data treatment process on-the-go. Here, we provide a pipeline for cleaning up fungal ITS metabarcoding data generated on Illumina MiSeq sequencers with a paired-end option. Instead of creating a complex, multifunctional pipeline we focused on the specific needs of fungal ecologists who intend to replace 454 with Illumina for their metabarcoding-based research, and who want to keep their data cleaning procedures up-to-date. The pipeline is assembled from simple, independent steps to facilitate further adaptations to the specific needs of the users and the implementation of future methodologies. We emphasize that users should be able to easily understand, modify, and error-check every data pruning step. The following considerations guided our pipeline assembly:

1. Best practices. We employed state of the art programs and adhered to existing recommendations for pruning fungal high-throughput data (Nilsson et al. 2011).
2. Flexibility. Users can modify and replace pruning steps. This is of particular importance considering the rapid changes in sequencing technology (e.g., increasing read lengths), the continuous development of new tools for particular problems, and peculiarities of specific molecular markers or data sets.
3. User friendliness. The steps in the pipeline were developed on an open-source Ubuntu Linux system, and its operation requires only basic knowledge of UNIX.

We found it important to provide detailed descriptions and explanations, as well as revision techniques for each step to help users understand individual procedures, and avoid mistakes. We think that security checks are essential when setting up a data cleaning pipeline. Users might be more inclined to leave out the control steps with a wrapped pipeline. This pipeline will make sequence cleanup more efficient for researchers with basic bioinformatics background and accelerate research in the field of fungal community ecology.

## Materials and Methods

In the following, we use the example of a fungal ITS rDNA data set to describe molecular methods and pipeline development.

### Molecular methods

We collected 96 soil samples from a low-input meadow located at Flörsheim, Germany (N50° 0′ 26.482′′, E8° 23′ 58.502′′). We extracted total DNA from 300 mg of soil using the FastDNA SPIN Kit for Soil (MP Biomedicals, Santa Ana, CA). We PCR amplified the ITS2 rDNA region with the primers ITS3_KYO2 and ITS4_KYO3 (Toju et al. 2012). We used two annealing temperatures (51°C and 55°C) and three replicated PCRs for each annealing temperature to account for the stochasticity of PCR reactions (Schmidt et al. 2013). Amplifications were carried out in a total volume of 20 *μ*L using 10 ng of DNA, 4 *μ*L of HOT MOLPol Blend Master Mix (Molegene, Germany), and 0.8 *μ*L (10 *μ*mol/L) of each primer. PCR conditions were 15 min at 95°C, followed by 30 cycles of 30 sec at 95°C, 30 sec at either 51°C or 55°C, and 30 sec at 72°C, and final elongation for 5 min at 72°C. PCR products were purified with Agencourt AMPure XP SPRI magnetic beads (Beckman Coulter, Brea, CA). We labeled the primers with 8 bp long tags (Kozarewa and Turner 2011) to identify samples after multiplexed sequencing. We used the same labels for forward and reverse primers. Labeling-PCRs were carried out in a total volume of 30 *μ*L using 20 ng of purified PCR product, 6 *μ*L of HOT MOLPol Blend Master Mix, and 1 *μ*L (10 *μ*mol/L) of each labeled primer. PCR conditions for this reaction were 15 min at 95°C, followed by six cycles of 30 sec at 95°C, 30 sec at 52°C and 30 sec at 72°C, and final elongation for 5 min at 72°C. Amplicons were visualized with gel electrophoresis. After purification with Agencourt AMPure XP SPRI magnetic beads we normalized and pooled the PCR products. We sequenced three amplicon pools on the Illumina MiSeq platform using the paired end (2 × 250 bp) option at the University of Minnesota Genomics Center. The fragment length distribution of the amplicons was analyzed on Agilent Bioanalyzer assays at the University of Minnesota Genomics Center before sequencing.

### Pipeline dependencies

This pipeline was elaborated and run on an Ubuntu 12.04 system. The following programs, scripts and data bases were used:

#### Programs

1. PANDAseq (Masella et al. 2012, https://github.com/neufeld/pandaseq/wiki/PANDAseq-Assembler).
2. fqgrep (https://github.com/indraniel/fqgrep).
3. Fastx Toolkit (http://hannonlab.cshl.edu/fastx_toolkit/).
4. FungalITSextractor (Nilsson et al. 2010, http://emerencia.org/FungalITSextractor.html).
5. MEGAN v4 (Huson et al. 2011, http://ab.inf.uni-tuebingen.de/software/megan/).
6. USEARCH v7.0.1001 (Edgar 2010, http://drive5.com/uparse/).
7. BLAST v2.2.27+ (Altschul et al. 1997).

#### Scripts

1. Reads_Quality_Length_distribution.pl (Supplementary Material).
2. remove_multiprimer.py (Supplementary Material).
3. demultiplex.sh (Supplementary Material).
4. rename.pl (Supplementary Material).
5. fasta_number.py (USEARCH v7, http://drive5.com/python/).
6. uc2otutab.py (USEARCH v7, http://drive5.com/python/).

#### Data bases

1. GenBank (http://www.ncbi.nlm.nih.gov/genbank/).
2. UNITE (Abarenkov et al. 2010a).

#### Data bases

(Supplementary Material, http://dx.doi.org/10.12761/SGN.2014.2)

1. subsampled Illumina MiSeq fastq files (exp02pool02_S1_L001_R1_001.fastq, exp02pool02_S1_L001_R2_001.fastq, each 50,000 paired-end, 250 bp-long reads).
2. primers (primers.txt).
3. Comma separated value (CSV) files containing sample names and labels + primers (forward_labels.csv, reverse_labels.csv).

#### Notes

The scripts and commands used here use the following file extensions: .fasta for fasta, .fastq for fastq, .csv for CSV. The scripts can be edited to comply with different extension naming.

All commands and programs should be added to the system path, or they should be present in the same folder from where they are called.

## Pipeline for processing Illumina metabarcoding reads

Processing fungal metabarcoding data is prone to complications and errors. It is important to consider the characteristics of the barcoding fragment, the sequencing instrument (e.g., error type and frequency), and the particular problems that come from PCRs and sample multiplexing. Our pipeline consists of procedures aimed at (1) assuring sequence quality; (2) assembling paired-end reads; (3) reliable demultiplexing; (4) the separation of informative barcode fragments from low-variability fragments (“ITS extraction”); and (5) the identification of fungal OTUs. Sequence quality is assured via the removal of low-quality reads, plausible chimeras, and clustering at thresholds higher than the expected sequencing error frequency. Paired-end assembly of the forward- and reverse-sequenced reads is important for the recovery of the complete ITS2 fragment, and it also allows reliable demultiplexing with multiplexing labels located on both ends of the fragments. It is important (and specific to fungal barcoding) that the barcode is a noncoding DNA fragment, with highly variable (i.e., taxonomically informative) and rather conserved parts. It is recommended for both clustering, and BLAST searches that only the highly variable regions are used (Nilsson et al. 2010). Reads are generally grouped into OTUs using sequence similarity (clustering) before ecological analyses. It is important to retain only reads that likely originate from the target organisms (but not from, e.g., plants) before proceeding with ecological analyses.

We defined the order of steps with regard to practical considerations. For example, it is practical to do the paired-end assembly immediately before removing primer artifacts (i.e., as step 2 instead of step 3 in the pipeline), otherwise an extra step is needed to ensure that the order of reads in the two fastq files is preserved. In general, we recommend the following:

### Quality filtering

Raw read pairs are filtered for an average read quality threshold with a script provided here. It is important to preserve the order of the reads in both forward and reverse read files: the paired-end read assembler needs corresponding read orders in both files.

perl Reads_Quality_Length_distribution.pl -fw forward_reads.fastq -rw reverse_reads.fastq -sc 33 -q26 -l 150 -ld N

*Explanation:* -sc Illumina phred score format, -q mean quality threshold, -l reads shorter than this number will be discarded, -ld give read length distribution.

*Output:* forward and reverse fastq files with quality-filtered sequences. If a sequence is removed from either of the files due to low quality, its pair is also removed from the other file.

*Recommended checks*: fastq files show the desired improvement (use, e.g., FastQC for easily checking this, http://www.bioinformatics.babraham.ac.uk/projects/fastqc/); read loss is low.

### Paired-end assembly

We assembled paired-end reads with PANDAseq (Masella et al. 2012). The program corrects mismatching bases in the overlapping region according to the basecall with the higher quality.

pandaseq-f forward_reads.fastq-r reverse_reads.fastq-F N -o 5 > paired_assembled.fastq

*Explanation:* -F preserve fastq format, -N remove reads with unknown nucleotides, -o minimum read overlap between forward and reverse.

*Output:* fastq file containing the paired-assembled sequences. The program runs only if the sequence order in the forward and reverse fastq inputs is the same.

*Recommended checks:* sequence loss is low; both forward and reverse primers are present in the expected places; a random blast of some reads gives reasonable hits; the fragment length distribution (use, e.g., FastQC for this) resembles what was expected from the Agilent High Sensitivity DNA assay chip results from the initial sequencing library QC (if available).

### Remove primer artifacts

We observed that sequences may contain multiple primer occurrences. Primers were sometimes found in the middle of the sequence, sometimes multiple times at the ends of the sequences. Sequences with such primer artifacts had lengths similar to the expected length of the ITS2 fragments. They were not observed with gel electrophoresis, and with Agilent Bioanalyzer assays. These sequences should be removed with a script supplied here. The script cannot handle nucleotide ambiguities in the primer sequence.

python remove_multiprimer.py -i input.fastq -o output.fastq -f <forwardPrimerSequence> -r <reversePrimerSequence>

*Output:* fastq file with sequences that do not contain primer multimers.

*Recommended checks:* search for the location of primers in the sequence data, for example with the command *grep*.

### Reorient reads to 5′-3′

Paired-end reads from the Illumina platform are randomly attached to the sequencing lane, so that the output files contain approximately 50% reads in each direction. These reads must be reoriented in 5′-3′ direction for all downstream steps. This can be done using grep-type commands to separate reads containing the forward and reverse primers. Some grep-type commands allow mismatches in the search strings. We use fqgrep, which is specifically developed for manipulating fasta and fastq textfiles. Then 3′-5′ reads can be easily reverse complemented (we use a Fastx Toolkit command, fastx_reverse_complement for this).

fqgrep -mN -p 'forward_primer_sequence' -e paired_assembled_good.fastq > good_5-3.fastq

fqgrep -mN -p 'reverse_primer_sequence' -e paired_assembled_good.fastq > good_3-5.fastq

*Explanation:* -m allow N mismatches, -p Pattern of interest to grep, -e allow logical expressions (e.g., for use with ambiguous sites [AGCT]).

*Output:* two fastq files, one containing reads sequenced in 5′-3′ direction, the other containing reads sequenced in 3′-5′ direction during the first sequencing run.

fastx_reverse_complement -Q33 -i good_3-5.fastq >> good_5-3.fastq

*Explanation:* -Q33 format of quality scores in the fastq (if needed).

*Output:* fastq file with all reads reoriented into 5′-3′ direction.

*Recommended checks:* randomly check the beginnings and ends of sequences for presence of correct primers. This can be done using the standard commands less, grep, head, tail, or in a sequence alignment viewer (SeaView is a simple and fast option on Linux, Gouy et al. 2010). The fastq file needs to be converted to fasta with for example, the fastq_to_fasta of the FASTX Toolkit (http://hannonlab.cshl.edu/fastx_toolkit/index.html).

### Demultiplexing

We retain only those reads that contain a perfectly matching primer + label combination on both ends. We use a script that relies on fqgrep (https://github.com/indraniel/fqgrep). The script can be modified according to the fqgrep manual to allow more complicated search patterns and fastq output. Currently the output files are in fasta format.

bash demultiplex.sh forward_labels.csv reverse_labels.csv 5-3_oriented.fastq

*Explanation:* forward_labels.csv: CSV (comma separated value) file containing sample names and the 5′-3′ orientation label + primer sequences (see sample files); reverse_labels.csv: CSV file containing sample names and the 3′-5′ orientation label + primer sequences (see sample files); 5-3_oriented.fastq: sequences reoriented to 5′-3′ direction.

*Output:* separate fasta files, each corresponding to a sample.

*Recommended checks:* check random demultiplexed samples for the presence of labels + primers at the expected ends; check whether names correspond to primer combinations; all expected samples are retained; read number differences among samples are not substantial.

### Pool files and remove primers and labels

The name of the samples is inserted into the headers of the fasta reads. Once the sequence headers contain the sample names, the samples can be pooled, and the primers and labels can be removed. We use a command from the Fastx Toolkit to trim labels and primers.

perl rename.pl

*Explanation:* the script introduces the file names into the headers of the fasta reads. The script has to be run in the folder containing the sample fasta files.

*Output:* separate fasta files, each corresponding to a sample. The file names (sample names) are included in the header of each sequence.

cat *.fasta >> combined_samples.fasta

*Explanation:* combines samples.

*Output:* fasta file with pooled sequences from each sample. The sample identity is preserved in the sequence headers.

fastx_trimmer -f 27 -i combined_samples.fasta -o head_trimmed.fasta

fastx_trimmer -t 26 -i head_trimmed.fasta -o trimmed.fasta

*Explanation:* -f first base to keep, -t trim N reads from the end of the sequences (these two steps cannot be combined in the fastx_trimmer; our primer + label combinations are 26 bp long).

*Output:* fasta file with forward and reverse primers, and multiplexing labels removed.

*Recommended checks:* Check the beginning and end of the reads to ensure that primers were removed completely, for example, in SeaView; check if combined read numbers make sense.

### Extract fungal ITS

Internal transcribed spacer amplicons contain conserved regions of the SSU (small rRNA subunit gene), 5.8S and LSU (large rRNA subunit gene), where the primers are located. If not removed, these conserved regions may bias clustering and BLAST searches, because they increase similarity among sequences (Nilsson et al. 2010). The FungalITSextractor (Nilsson et al. 2010) extracts ITS1 and/or ITS2 from the reads, or discards reads if they do not match the structural ITS model. The script shortens long fasta headers, so it is important to remove unnecessary information from the headers before extracting the ITS. The redundant information generally refers to the properties of a sequencing run. The truly sequence-specific parts of a header are the physical coordinates of the DNA fragment cluster on the sequencing lane. The *sed* UNIX command removes redundant information from the sequence headers. The time required for ITS extraction can be reduced if the input file is split into several parts which are run separately, and the results are combined again.

sed 's/<redundant information>//g' trimmed.fasta > trimmed_named.fasta

perl FungalITSExtractor.pl

*Explanation:* The file with the unique sequence headers has to be copied into the indata folder of the FungalITSExtractor tool. The results are in the outdata folder.

*Output:* fasta files with extracted ITS sequences in the outdata folder of the FungalITSExtractor. Sequences not corresponding to the ITS sequence model are stored in separate files.

*Recommended checks:* aligning a number of reads in some samples does not show conserved alignments at the beginning and end of the reads; randomly picked extracted ITS sequences give positive (5′-3′) hits in the UNITE/GenBank. Check the headers of the resulting fasta file to ensure that it preserves enough information on sequence identity.

### Similarity clustering

The clustering is based on the uparse pipeline (Edgar 2013) of the USEARCH v7 (Edgar 2010). The steps include (1) grouping of replicate sequences; (2) sorting sequences according to decreasing abundance; and (3) OTU identification and de-novo chimera filtering.

usearch -derep_fulllength ITS2.fasta -output derep.fasta -sizeout

*Explanation:* -sizeout adds the number of replicate sequences into the header of their representative sequence.

*Output:* fasta file with unique ITS sequences.

usearch -sortbysize derep.fasta -output sorted.fasta -minsize 2

*Explanation:* -minsize removes representative sequences that are present less than *n* times. We generally remove singletons during this step.

*Output:* fasta file without singletons (or doubletons, tripletons, etc., by choice).

usearch -cluster_otus input_file.fasta -otus output_file_otus.fasta -otuid 0.97

*Explanation:* -otuid specifies the clustering threshold.

*Output:* fasta file with the centroid sequences of the clusters generated at the specified similarity threshold.

*Recommended checks:* Follow read loss by summarizing the size information from the headers of the cluster-representative sequences, for example

grep ">" otus.fasta | sed "s/size=/,/g" > headers.csv

Open headers.csv in a spreadsheet program and sum the read numbers.

### Reference-based chimera filtering

We perform a second chimera filtering step using USEARCH v7 (Edgar 2010). The database of the plausible parent sequences is generated from the UNITE fungal ITS database (Abarenkov et al. 2010a).

usearch -makeudb_usearch UNITE_input_database. fasta -output UNITE.udb

*Explanation:* ITS fasta sequences from UNITE are converted into a reference database.

*Output:* USEARCH-formated database file for reference-based chimera filtering.

usearch -uchime_ref otus.fasta -db UNITE.udb -nonchimeras otus_good.fasta -chimeras otus_chim.fasta -strand plus

*Explanation:* -strand plus: the filtering is correct only if DNA sequences are in 5′-3′ direction, and if this is explicitly specified.

*Output:* fasta files containing sequences deemed nonchimeric or chimeric.

*Recommended checks:* Follow chimeric sequence numbers: generally chimera formation is not extensive, although some chimeras are found. A BLAST search of randomly picked chimeric sequences suggests that these are indeed chimeras. False positives are possible. Nilsson et al. (2012) present useful ideas to identify chimeras with BLAST searches.

### Identify fungal OTUs

Primers used for fungi may (and do) amplify sequences from nontarget organisms; for example, plants. OTUs that do not belong to the target taxa must be discarded. We BLAST OTU representative sequences against the GenBank nucleotide database (nt). Although not specifically curated for fungal ITS sequences (in contrast to the fungal ITS-specific UNITE (Abarenkov et al. 2010a), blasting against the nt will give information also about the proportion of diverse taxonomic groups that may have been co-amplified with fungal target sequences. We use this step to select OTUs of fungal origin for downstream ecological analyses. We do not attempt taxonomic assignments of the OTUs in this step. The most current nt database can be obtained with an NCBI download script (update_blastdb.pl, part of the BLAST+). We use MEGAN (Huson et al. 2011) to parse the BLAST results, and to retain the ITS sequences of the fungal OTUs.

blastn -db /database/GenBank/nt -query input.fasta -outfmt 5 -out output.xml -num_threads=N

-evalue 0.001

*Explanation:* -outfmt 5 specifies xml as output format, -num_threads allows to use multiple processors, -help gives a detailed list of options.

The xml, and the blasted fasta file, is imported into MEGAN. The lowest common ancestor assignments depend on several options, our choices for Illumina paired-end reads are minimum reads 1, minimum score 170, upper percentage 5, no minimum complexity, no min complexity (0). We uncollapse all branches, select Fungi, and from the Select menu select Subtree. Reads should be exported from the File menu (File/Export/Reads).

*Output:* xml file containing the BLAST hits of the OTU centroid sequences. rma file containing the parsed BLAST results. fasta file containing the exported fungal OTU sequences.

*Recommended checks:* there are no issues with the fasta header formatting of the OTUs when inspecting taxon assignments; the number of OTUs that MEGAN shows as fungi corresponds with the number of OTU sequences exported into a fasta file; count number of reads in the resulting fungal_otus.fasta file and compare with the MEGAN counts; check if sequence headers are complete.

### Fungal OTU abundance table

The generation of the fungal abundance table is based on python scripts supplied with USEARCH v7 and includes the following steps: (1) label OTU-representative sequences according to a pattern (here: OTU_1, OTU_2, etc.); (2) map original, not clustered reads (here: ITS2 sequences generated by FungalITSExtractor) against the fungal OTU-representative sequences; (3) specify the sample information in the mapping results; and (4) generate the OTU table from the mapping results.

python fasta_number.py fungal_otus.fasta OTU_ > fungal_otus_numbered.fa

usearch -usearch_global ITS2.fasta -db fungal_otus_numbered.fa -strand plus -id 0.97 -uc fungal_readmap.uc

sed -i 's/REV/barcodelabel=REV/g' fungal_readmap.uc

*Explanation:* The name of the samples has to be specified in the readmap file (barcodelabel=). Our sample naming scheme allows for a simple sed-based modification of the readmap. Alternatively, the USEARCH v7 script fastq_strip_barcode_relabel.py may also be used to specify the sample names.

*Output:* fasta file with numbered OTU sequences. Text file (readmap.uc) containing the results of OTU centroid sequence mapping against the original ITS file.

python uc2otutab.py fungal_readmap.uc > fungal_otu_table.txt

*Output:* Text file (tab-delimited) containing the OTU abundance table of samples.

*Recommended checks:* Check read sums in the abundance table according to samples/OTUs.

## Results

**Figure 1:**
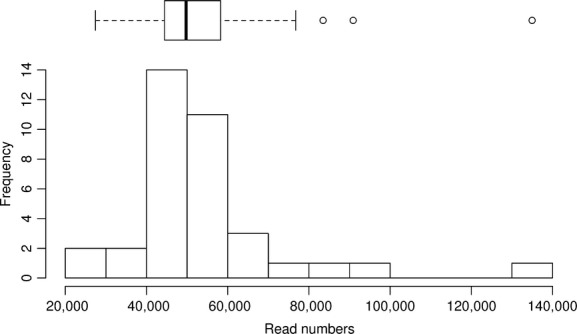
Frequency of soil samples in relation to the number of Illumina MiSeq reads allocated to each sample. The majority of samples contained between 40,000 and 70,000 Illumina MiSeq reads.

**Figure 2:**
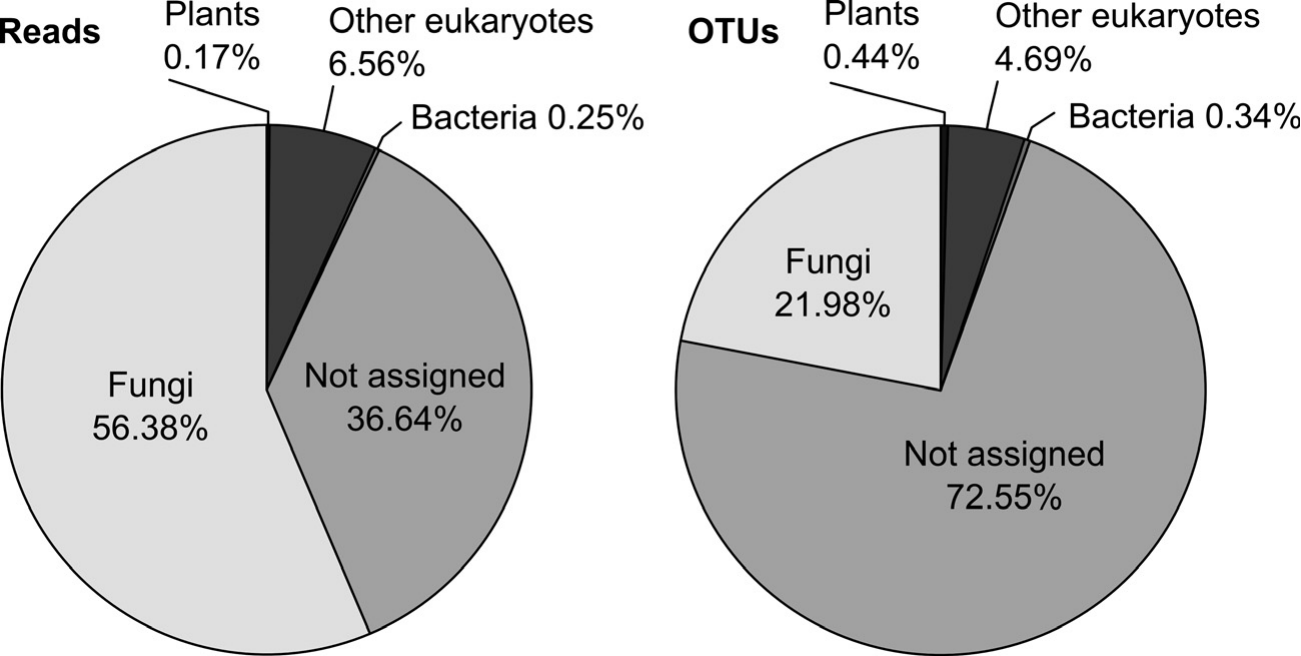
Distribution of Illumina MiSeq reads and 97% OTUs across different taxonomic groups. Approximately, 5.9 million reads are assigned to fungi (from a total of >10 million reads). From a total of 14,636 OTUs 3208 could be assigned to fungi.

The three sequencing runs resulted in ∼40 million paired-end reads (Table 1). Reads were relatively evenly distributed among the 80 multiplexed samples (Fig. 1). The pruning of the raw sequence data resulted in considerable read losses: ∼30 million reads were lost during paired-end assembly, reorientation, demultiplexing, and chimera checking, and only ∼10 million reads were considered correct ITS2 sequences after ITS extraction. Most reads were lost during the demultiplexing step. PhiX DNA fragments were added to the sequencing reactions as a standard practice during the Illumina sequencing of amplicons, but the PhiX sequences were removed during the sequence postprocessing already by the sequencing facility. Thus, it is not the presence of the PhiX fragments that causes the read losses observed during demultiplexing. Finally, about 6 million of the pruned reads were assigned to fungi, while most of the remaining sequences could not be taxonomically assigned (Fig. 2).

**Table 1:** Steps of the Illumina metabarcoding data denoising pipeline using a fungal ITS rDNA example file. We present decreasing read/cluster numbers, approximate computing times, and computing infrastructure for each step. Computing times are based on the example data file run on a standard desktop computer with two processors and 4GB RAM or a massive RAM machine with 48 processors and 512GB RAM

Pipeline step	Program	Files	Read numbers	Cluster numbers	Time	Computer	Processors
Raw sequence data		Pool 1 forward	14.940.845				
		Pool 1 reverse	14.940.845				
		Pool 2 forward	11.209.268				
		Pool 2 reverse	11.209.268				
		Pool 3 forward	13.946.058				
		Pool 3 reverse	13.946.058				
1. Quality filtering	Script (Supplements)	Pool 1 forward	13.433.309		Up to 1 h	Desktop computer	2
	Reads_Quality_Length_distribution.pl	Pool 1 reverse	13.433.309				
		Pool 2 forward	9.998.878				
		Pool 2 reverse	9.998.878				
		Pool 3 forward	13.044.704				
		Pool 3 reverse	13.044.704				
2. Paired-end assembly	PANDAseq	Pool 1	12.341.403		Up to 1 h	Desktop computer	2
		Pool 2	9.314.737				
		Pool 3	12.134.242				
3. Remove primer artifacts	Script (Supplements)	Pool 1	11.255.037		Minutes	Desktop computer	2
	remove_multiprimer.py	Pool 2	8.520.491				
		Pool 3	11.153.448				
4. Reorient reads to 5′-3′	fqgrep	Pool 1	9.061.462		Up to 1 h	Desktop computer	2
		Pool 2	8.155.112				
		Pool 3	10.539.993				
5. Demultiplex	Script (Supplements)						
(A) forward labels	demultiplex.sh	Pool 1	8.851.827		Up to 1 h	Desktop computer	2
		Pool 2	8.053.268				
		Pool 3	9.903.834				
(B) reverse labels		Pool 1	3.297.016		Up to 1 h	Desktop computer	2
		Pool 2	2.957.182				
		Pool 3	3.949.934				
6. Pool files, remove primers and labels	rename.pl	Pool 1, 2 and 3 combined	10.203.752		Minutes	Desktop computer	2
7. Extract fungal ITS	FungalITSextractor	Pool 1, 2 and 3 combined	10.093.751		Several days	Computer cluster	50
8. Similarity clustering							
(A) Remove replicate sequences	UPARSE	Pool 1, 2 and 3 combined		4.869.466	Minutes	Desktop computer	2
(B) Sort sequences by abundance	UPARSE	Pool 1, 2 and 3 combined		560,678	Minutes	Desktop computer	2
(C) Cluster OTUs	UPARSE	Pool 1, 2 and 3 combined		14,781	Up to 1 h	Desktop computer	2
9. Reference-based chimera filtering	UPARSE	Pool 1, 2 and 3 combined		14,636	Up to 1 h	Desktop computer	2
10. Identify fungal OTUs							
(A) BLAST	BLAST	Pool 1, 2 and 3 combined		14,636	Several hours	Computer cluster	25
(B) Assign fungal reads	MEGAN	Pool 1, 2 and 3 combined		3208	Minutes	Desktop computer	
11. Fungal OTU abundance table	UPARSE	Pool 1, 2 and 3 combined	5.964.069	3208	Several hours	Desktop computer	2

The ∼10 million pruned ITS2 reads were clustered into 16,623 operational taxonomic units (OTUs) at 97% sequence similarity by the heuristic clustering algorithm of USEARCH v7. Of these, 1842 OTUs were deemed chimeric by the USEARCH-based de-novo chimera filtering, and 145 by the reference-based chimera filtering. The two chimera-checking steps suggest that ∼11.95% of all OTUs were chimeric. Of the remaining 14,636 OTUs, only 3208 were assigned to fungi (Fig. 2). Most OTUs could not be assigned to any known taxon.

## Discussion

The pipeline presented here fills an important gap as the first collection of practical steps for cleaning fungal ITS metabarcoding data generated as paired-end reads on Illumina MiSeq sequencers. In our opinion the Illumina MiSeq platform is a likely successor of 454 pyrosequencing in the metabarcoding of fungi, at least until the quality issues of the IonTorrent platforms are solved (Brown et al. 2013). Given the importance of fungi in almost every natural system, we expect rapid advances on this field.

We benchmarked our pipeline with a large ITS2 metabarcoding dataset generated as paired-end, 2 × 250 bp long reads during three MiSeq runs. We experienced considerable read losses during data cleanup: from the original ∼40 million reads only ∼6 million was suitable for downstream ecological analyses as high-quality fungal reads. About 2.8 million sequenced reads contained multiple primer occurrences, an error type that we were unaware of from the literature. Most sequences were lost during the reorientation and demultiplexing steps: ∼20 million reads did not contain the correct primer and/or label sequences on both ends. We note that we were conservative by not allowing any mismatches in the primer or label sequences, and this likely contributed to the substantial read losses during this step. We were also conservative with chimeras by applying two (a *de-novo* and a reference-based) chimera filtering steps. The separation of the variable ITS region from the conversed surrounding fragments (an important step in processing hypervariable ITS data) was the most time-consuming step that demanded hundreds of processor hours. The time required by this step can be substantially reduced by performing the ITS extraction after the de-replication of sequences (Table 1, step 8A).

Our pipeline considers all data cleanup steps recommended for fungal metabarcoding (Nilsson et al. 2011). It also considers the specifics of the fungal metabarcode and the characteristics of the Illumina sequencing platform. Finally, the pipeline addresses not only the known issues of metabarcoding (e.g., low-quality reads, chimeras, possible multiplexing label switching), but also a new problem we encountered: the multiple occurrences of the barcoding primers in some sequences. The pipeline is highly modular and easily modifiable with basic UNIX knowledge. We also emphasize its transparency, as users should understand the way each cleaning step is carried out. We included “revision techniques” for each step, as we found it important to continuously check the correctness of data generated in each step, and to correct the performance of each program. This is especially important during the “testing” phase of the pipeline, i.e., when modular steps need replacement, or when the experimental design requires alteration. Many of these checks may seem obvious for researchers experienced in data cleanup, but our own experience shows that they considerably help inexperienced users to understand, perform and verify each step. We refrained from providing a wrapper script for the entire pipeline, but all steps presented here can be easily wrapped up in simple bash scripts, or in workflow engines such as Snakemake (Köster and Rahmann 2012). Wrapping steps is up to the users, and in our opinion it should be done only after being confident that every data processing step provides reliable results.

We see this pipeline as a user-friendly and loose collection of recommendations and practical instructions for analyzing fungal Illumina-based ITS data, rather than a final and standalone product. We emphasize the adaptability of the pipeline to ever-changing user needs and technological advances. We expect that our pipeline will be modified in the future to deal with new technological/methodological challenges. Every modular step can be replaced with alternatives; for example, Trimmomatic for quality filtering of paired-end data (Lohse et al. 2012), the ITSx (Bengtsson-Palme et al. 2013) for ITS extraction, or GramCluster (Russell et al. 2010) for a nondistance-based clustering. Confirmation for the user friendliness of this pipeline came from participants of a Masters' course in ecology and evolution at the Goethe University of Frankfurt. Students who had never processed this type of data before and were largely inexperienced in bioinformatics had no difficulties in learning and independently applying the pipeline after a few hours of hands-on training.

According to our experience metabarcoding pipelines should be collections of easy-to-adapt simple scripts and practical computer commands, rather than complex tools. Users should have maximum understanding and control over the pruning of their data to avoid mistakes. Although complex standalone pipelines have the advantages of providing relatively generalized solution to a range of problems, they lag behind in the specificity demanded by individual research groups and particular experiments. We hope that our pipeline will allow researchers to analyze their paired-end Illumina fungal ITS metabarcoding data more readily.

## Data Accessibility

We deposited sequence data and scripts as digital objects at http://dx.doi.org/10.12761/SGN.2014.2. The same scripts and sample files are also provided as Supplementary materials.

## Supporting Information

**Data S1.** Sample files: exp02pool02_S1_L001_R1_001.fastq; exp02pool02_S1_L001_R2_001.fastq; primers.txt; forward_labels.csv; reverse_labels.csv.

**Data S2.** Scripts: Reads_Quality_Length_distribution.pl (Supplementary Material); remove_multiprimer.py (Supplementary Material); demultiplex.sh (Supplementary Material); rename.pl (Supplementary Material).
